# Supplementary material for: Inhibition within a premotor circuit controls the timing of vocal turn-taking in zebra finches
Source: Nat Commun. 2020 Jan 10;11:221. doi: 10.1038/s41467-019-13938-0 (PMC6954284; doi:10.1038/s41467-019-13938-0)
Supplement: Supplementary file 1 — Supplementary Information [file 41467_2019_13938_MOESM1_ESM.pdf]

Supplementary Information

Inhibition within a premotor circuit controls the timing of vocal turn-taking in zebra finches

Jonathan I. Benichov, Daniela Vallentin*

*Correspondence to: daniela.vallentin@orn.mpg.de

This PDF file includes:

Supplementary Figs. 1–4

Captions for Supplementary Video 1

Other Supplementary Materials for this manuscript include the following:

Supplementary Video 1

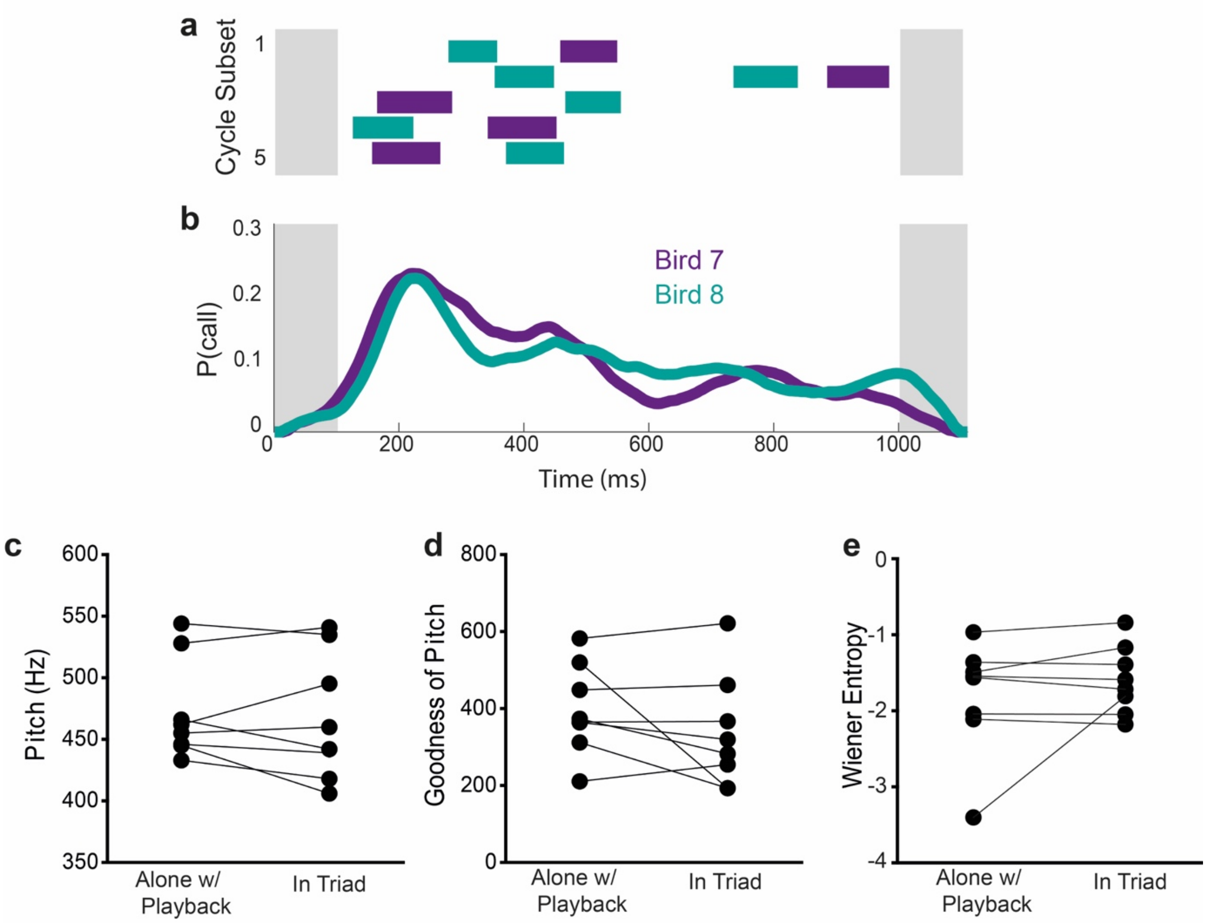

Supplementary Fig. 1 Call Coordination and Call Acoustic Structure in Vocal Triads. **a** A subset of sequential call cycles demonstrating an alternating response pattern between Bird 7 (purple) and Bird 8 (turquoise) during the vocal triad condition. **b** Call response probability distributions for Birds 7 and 8 for a full 10-minute session when in triad with call playbacks (in grey). Peak response latencies remain similar, although the pair's alternating call sequences result in lower than expected simultaneous calling. **c** Mean call pitch for n = 8 birds while alone with playbacks vs. in the triad condition (Alone w/playbacks = 472 ± 40 Hz, in triad = 467 ± 51 Hz, Wilcoxon sign rank test, p = 0.4609). **d** Harmonic structure of calls as measured by mean Goodness of Pitch for n = 8 birds while alone with call playbacks vs. in the triad condition (mean (alone w/playback) = 397 ± 118, (in triad) = 337 ± 146, Wilcoxon signed rank test, p = 0.3125). **e** Average Wiener Entropy of calls for n = 8 birds while alone with call playbacks vs. in triad condition (mean(alone w/playback = -1.81 ± 0.74, (in triad) = -1.59 ± 0.45, Wilcoxon signed rank test, p = 0.8438). Note: Each bird's average was calculated from 54-243 calls for all acoustic features.

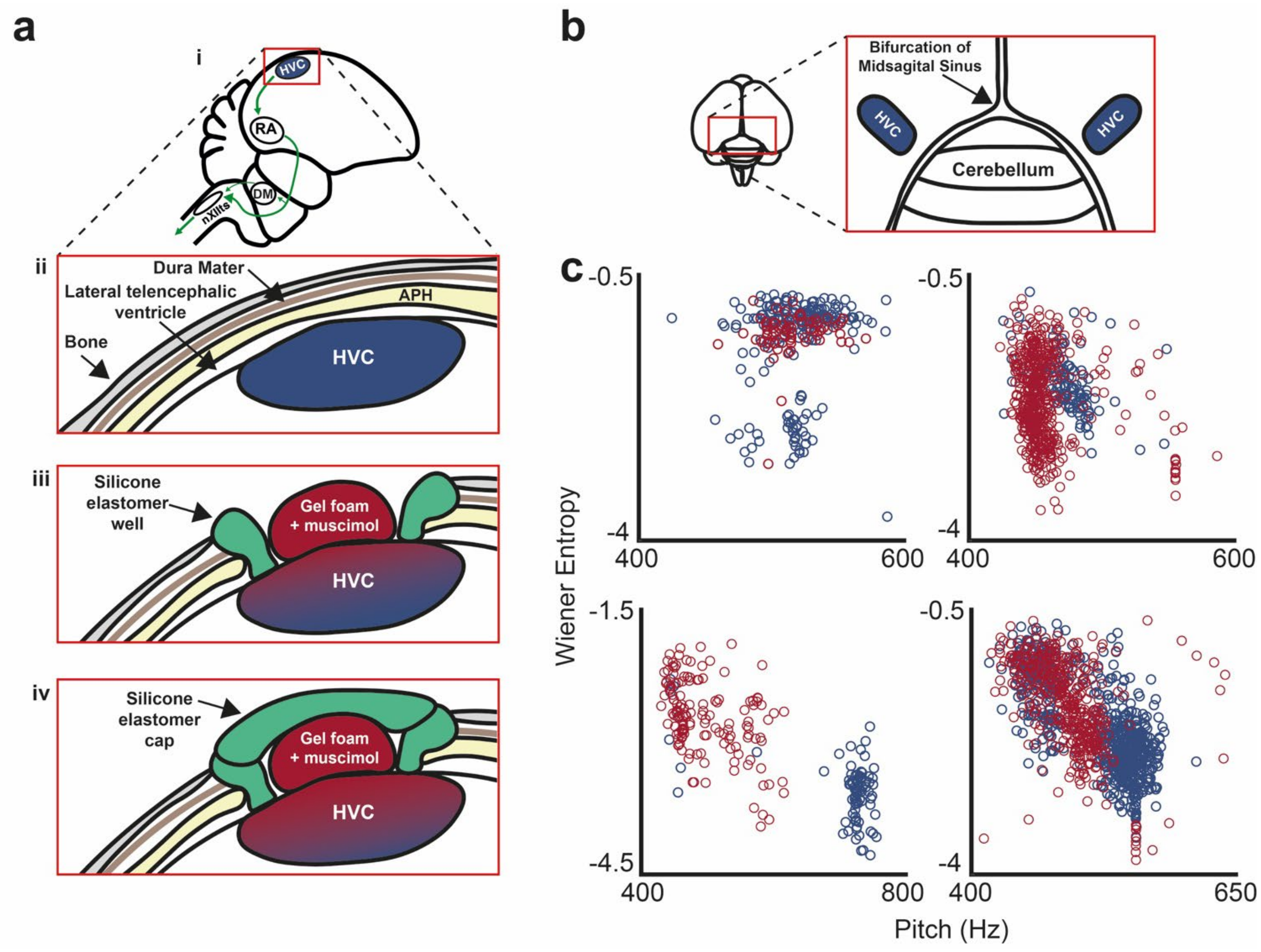

Supplementary Fig. 2 a) Schematic of pharmacological infusion from the dorsal surface of HVC. i) Illustration of sagittal section of zebra finch vocal motor pathway (RA:robust nucleus of the arcopallium; DM: dorsomedial part of the intercollicular nucleus; nXIIts: 12th cranial (tracheosyringeal) nerve. ii) Bone, dura mater, APH (area parahippocampalis), and the lateral telencephalic ventricle are situated above HVC. iii) A craniotomy is cut, dura mater and APH are resected, and a silicone elastomer well is created around the perimeter of the craniotomy. A gel foam sponge soaked in 40°C 5mM muscimol is placed directly on the surface of HVC. iv) A silicone elastomer cap seals the well and the animal is returned to the recording chamber for behavioral testing. **b)** Schematic of a dorsal view of the zebra finch brain, indicating positions of bilateral craniotomies above HVC. **c)** Acoustic Features of Calls During Control and Muscimol Conditions. Changes in acoustic features of calls for 4 birds during infusion with musicmol (red) vs. saline controls (blue, as in Fig. 2g); entropy(control) = -2.52 ± 1.13, entropy(muscimol) = -2.21 ± 0.79, 2d-Kolmogorov-Smirnov test [1], $p < 0.01$ Bonferroni correction).

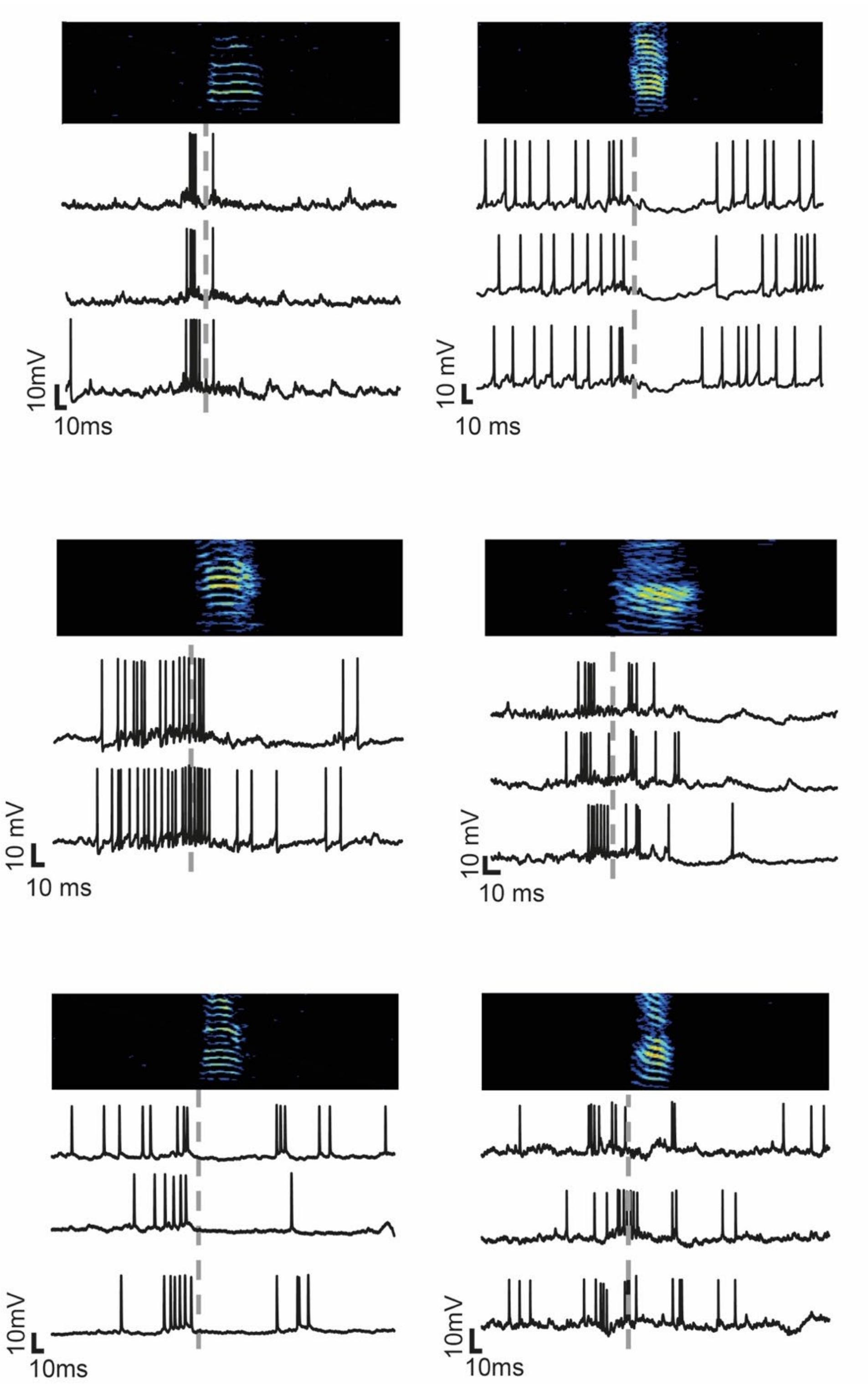

Supplementary Fig. 3 HVC Interneuron Activity During Call Production. Intracellular recordings of six HVC interneurons during multiple renditions of call production. Dotted grey lines indicate onsets of calls.

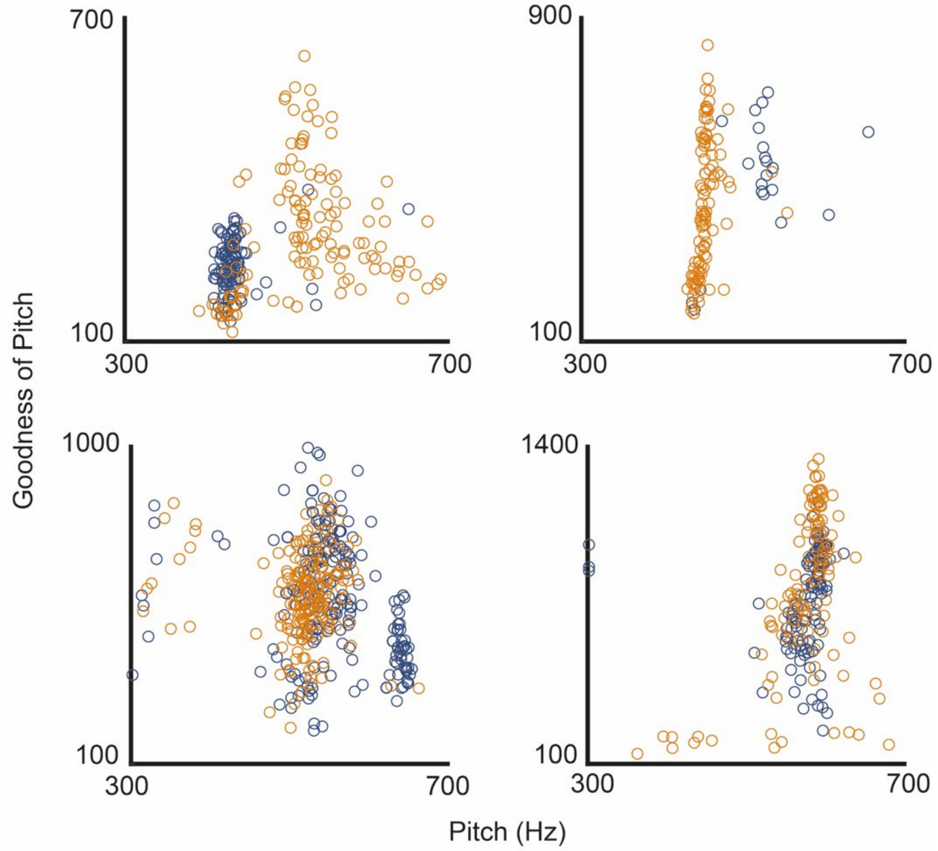

Supplementary Fig. 4 Acoustic Features of Calls During Control and Gabazine Conditions. Changes in acoustic features of calls for 4 birds during infusion with saline controls (blue) vs. gabazine (orange), (as in Fig. 5g), (2d-Kolmogorov-Smirnov test [1], $p < 0.01$ Bonferroni correction (4/5 birds)).

References

1. Peacock JA. Two-Dimensional Goodness-of-Fit Testing in Astronomy. *Mon Not R Astron Soc* **202**, 615-627 (1983).
