# Supplementary material for: Inhibition within a premotor circuit controls the timing of vocal turn-taking in zebra finches
Source: Nat Commun. 2020 Jan 10;11:221. doi: 10.1038/s41467-019-13938-0 (PMC6954284; doi:10.1038/s41467-019-13938-0)
Supplement: Supplementary file 3 — Description of Additional Supplementary Files [file 41467_2019_13938_MOESM3_ESM.pdf]

Description of Additional Supplementary Files

File Name: Supplementary Movie 1
Description: Zebra Finch Vocally Interacting with Call Playback During Saline Control and Gabazine Conditions. Concatenated video sequences when the bird responded to call playbacks during saline and gabazine conditions.
